# The Association Between Sleeping Pill Use and Metabolic Syndrome in an Apparently Healthy Population in Japan: JMS-II Cohort Study

**DOI:** 10.2188/jea.JE20200361

**Published:** 2022-03-05

**Authors:** Toshihide Izumida, Yosikazu Nakamura, Yukihiro Sato, Shizukiyo Ishikawa

**Affiliations:** 1 Division of Community Medicine, Kanazawa Medical University Himi Municipal Hospital, Himi, Toyama, Japan; 2 Division of Public Health, Center for Community Medicine, Jichi Medical University, Shimotsuke, Tochigi, Japan; 3 Internal Medicine, Kamiichi General Hospital, Nakaniikawa-gun, Toyama, Japan

**Keywords:** sleeping pills, metabolic syndrome, cardiovascular disease, epidemiology

## Abstract

**Background:**

Sleeping pills are widely used for sleep disorders and insomnia. This population-based study aimed to evaluate the association between the use of sleeping pills and metabolic syndrome (MetS) and metabolic components in an apparently healthy Japanese cohort.

**Methods:**

We examined baseline cross-sectional data from the JMS-II Cohort Study. The criteria for MetS and its components were based on The National Cholesterol Education Program Adult Treatment Panel III. Sleep habits including the sleep duration of the subjects and the frequency of sleeping pill use were obtained using The Pittsburgh Sleep Quality Index questionnaire. For different sleep durations, the association between sleeping pill use and MetS was assessed. Odds ratios (ORs) and their 95% confidence intervals (CIs) were estimated using multiple logistic regression models to quantify this association.

**Results:**

Our study included 6,153 individuals (mean age, 63.8 [standard deviation 11.2] years), and 3,348 (54.4%) among them were women. The association between sleep duration and MetS was an inverted J-shaped curve among sleeping pill users and a J-shaped curve among non-users. After adjustment for various confounders, less than 6 h of sleep among sleeping pill users was associated with increased rates of MetS (<6 h, OR 3.08; 95% CI, 1.29–7.34]). The frequency of sleeping pill use in individuals with short sleep duration showed a positive association with the prevalence of MetS and its components.

**Conclusions:**

Sleeping pill users with a short sleep duration had a 3-fold higher chance of having MetS than non-users with a short sleep duration.

## INTRODUCTION

Disturbed sleep, poor sleep quality (short and long sleep durations), and insomnia are not only linked to poor quality of life but also to metabolic syndrome (MetS) and cardiovascular diseases (CVDs); they might be important risk factors for atherosclerosis.^1^^–^^12^ The widely used therapies for sleep disorders and chronic insomnia include medications such as sleeping pills and cognitive behavioral therapy.^13^

Sleeping pills can exacerbate or inhibit atherosclerosis. The short-term use of sleeping pills improves sleep quality and extend sleep duration, resulting in a reduction in stress, and may indirectly affect metabolic function.^14^ The experimental human studies showed that benzodiazepine receptor agonists enhance the activity of gamma-aminobutyric acid (GABA) in the central nervous system and might directly reduce systolic and diastolic blood pressure (BP).^15^^,^^16^ The latency of rapid eye movement sleep, which is affected by the use of sleeping pills, was also associated with good glycemic control and decreased arterial wall thickening.^17^ Although these experimental studies might show potential benefits of short-term use of sleeping pills, the relationship among factors exacerbating or inhibiting atherosclerosis might be very complex in a clinical setting, and hence, it is important to elucidate the association between sleeping pill use and MetS in a real-world, population-based study.

Few cohort studies have reported the effects of sleeping pill use on MetS. However, contrary to the positive results of the experimental research, several clinical studies have reported that the use of sleeping pills may be associated with increased all-cause mortality and CVD incidence in the general population.^18^^–^^20^ There might be a significant discrepancy between the experimental science data and clinical data regarding the use of sleeping pills and atherosclerosis. Therefore, this study aimed to evaluate the association between the use of sleeping pills and MetS, considering sleep duration as an important control variable, in the Japanese population. This is the first study to conduct statistical analysis using the group divided by sleep duration as a control variable.

## METHODS

### Population

We examined the baseline cross-sectional data from the JMS-II Cohort Study. The design and main objective of our cohort study have been previously described.^21^ It included 6,436 Japanese individuals who underwent health screening tests conducted in accordance with the medical care system guidelines. This study aimed to investigate the risk factors for death due to atherosclerosis in the general Japanese population. It covered 13 rural districts in Japan, including the Shimotsuke, Kakara, Sue, Omori, Kamiichi, Wara, Takasu, Onabi, Nakatsu, Yame, Miwa, Ueno, and Saji areas, between April 2010 and December 2017. All the participants provided written informed consent prior to inclusion. We excluded individuals who had missing data for age; BP; levels of fasting glucose, triglycerides (TG), and high-density lipoprotein cholesterol (HDL-C); height; weight; waist circumstance (WC); medical history of myocardial infarction, stroke, and cancer; sleep habits; smoking history; history of alcohol consumption; education level; and marital status (*n* = 283).

### Measurements

A central committee, composed of the chief medical officers from all participating districts, developed a detailed manual for data collection. The participants’ height, weight, and WC were measured with 0.1 cm, 0.1 kg, and 0.1 cm as units of measurement, respectively. Body mass index (BMI) was calculated as weight (kg)/height (m^2^). Systolic BP and diastolic BP were measured using a fully automated sphygmomanometer (Omron HEM-759P; Omron Healthcare Inc., Kyoto, Japan), placed on the right arm of the subjects who had rested in the sitting position for 5 minutes before the measurement. Blood samples were collected after overnight fasting. An external laboratory (SRL, Tokyo, Japan) measured the levels of fasting glucose, total cholesterol (TC), TG, and HDL-C. Information about medical history and lifestyle were obtained using a self-reported questionnaire. Subjects were asked about their past and present illnesses and if they were taking medications for the same. Smoking status was classified as current-smoker, ex-smoker, or non-smoker.

Sleep habits were evaluated using the Pittsburgh Sleep Quality Index (PSQI).^22^^,^^23^ We divided the participants into three groups according to sleeping pill use (C6) of PSQI: participants who did not use sleeping pills during the past month (non-use group); participants who took sleeping pills <3 days per week (low-frequency-use group); and participants who took sleeping pills on ≥3 days per week (high-frequency-use group). Symptoms of depression and anhedonia were assessed using two questions: (1) “During the past month, have you been bothered by feeling down, depressed, or hopeless?” and (2) “During the past month, have you been bothered by little interest or pleasure in doing things?”^24^^,^^25^ This study was approved by the Institutional Review Board of Jichi Medical University (Tochigi, Japan, IRB No. G09-39 [G17-64 revised]).

### Definitions of metabolic syndrome

The criteria for MetS and its components were based on the modified National Cholesterol Education Program Adult Treatment Panel III (NCEP ATP III).^26^ The presence of at least three of the following five components was required for the diagnosis: abdominal obesity (WC [men, >102 cm; women, >88 cm]), elevated TG (≥150 mg/dL), low HDL-C (men, <40 mg/dL; women, <50 mg/dL), increased BP (systolic BP ≥130 mm Hg and/or diastolic BP ≥85 mm Hg), and elevated blood glucose (BG) (≥110 mg/dL). However, Japanese people are relatively petite; therefore, we adopted a WC threshold of >85 cm for men and >90 cm for women to define abdominal obesity, as proposed by the Japan Society for the Study of Obesity.^27^

### Statistical analysis

Baseline characteristics were summarized as means ± standard deviations (SD) for normally distributed continuous variables and as numbers and percentages for categorical variables. The TG values were highly distorted; thus, the data were expressed as medians and interquartile ranges and transformed into natural logarithms before statistical analysis. Differences between the sleeping pill user and non-user groups were compared using the unpaired *t* test and chi-squared test. Sleep duration was categorized into 5 categories (<6, 6 to 7, 7 to 8, 8 to 9, and ≥9) based on the hours of sleep per night. The relationship between sleeping pill use and MetS was assessed using multiple logistic regression analysis in the 5 sleep groups. A multivariable model was used and adjusted for age; sex; history of smoking; history of alcohol consumption; medical history of myocardial infarction, stroke, and cancer; marital status (yes or no); education status (<18 years or ≥18 years); depression symptoms (yes or no); and difficulty in initiating sleep (taking more than 1 hour to fall asleep at night [yes or no]). All statistical analyses were performed using SPSS version 22 (IBM, Chicago, IL, USA), and statistical significance was defined as *P* < 0.05.

## RESULTS

The subjects of our study were 6,153 individuals aged 28–95 years. The mean age was 63.8 years with an SD of 11.2 years, and 3,348 (54.4%) subjects were female. A summary of the baseline characteristics categorized by sleeping pill use is described in Table 1. Among the individuals included in our study, 858 (13.9%) used sleeping pills. Among the individuals using sleeping pills, 583 (67.9%) were in the high-frequency-use group. Older age, high TG levels, depression symptoms, and medical history of stroke and cancer were significantly more frequent in the sleeping pill user group than in the non-user group. No significant differences were observed in the prevalences of MetS, increased BP, impaired glucose tolerance, elevated TG levels, and abdominal obesity between these two groups.

**Table 1.** Baseline characteristics

	Sleeping pill user (*N* = 858)	Sleeping pill non-user (*N* = 5,295)	*P-value*
Male, *N* (%)	267 (31.1)	2,538 (47.9)	<0.001
Age, years	69.7 ± 10.2	62.9 ± 11.1	<0.001
BMI, kg/m^2^	22.8 ± 3.4	23.1 ± 3.3	0.026
Waist circumstance, cm	82.9 ± 9.5	82.8 ± 9.0	0.804
Fasting glucose, mg/dL	101.2 ± 20.1	100.2 ± 19.3	0.201
Total cholesterol, mg/dL	203.7 ± 32.8	205.0 ± 33.3	0.270
Triglyceride, mg/dL	100 (74, 136)	93 (67, 132)	0.003
HDL-C, mg/dL	58.7 ± 15.3	60.2 ± 14.8	0.004
Sleep duration, hours per night^a^	7.9 ± 1.6	7.4 ± 1.4	<0.001
Marriage status, *N* (%)^a^	625 (73.3)	4,470 (84.9)	<0.001
Education, *N* (%)^a^	410 (48.2)	3,577 (68.1)	<0.001
Depressive symptoms, *N* (%)^a^	322 (37.5)	1,210 (22.9)	<0.001
Difficulty initiating sleep, *N* (%)^a^	155 (18.1)	258 (4.9)	<0.001
Past medical history^a^			
Stroke, *N* (%)	36 (4.3)	129 (2.5)	0.003
Myocardial infarction, *N* (%)	29 (3.5)	133 (2.5)	0.128
Cancer, *N* (%)	80 (8.3)	332 (6.4)	0.032
Smoking^a^			
Current	66 (7.7)	729 (13.8)	<0.001
Ex	194 (22.6)	1,457 (27.5)	0.003
Never	598 (69.7)	3,109 (58.7)	<0.001
Current drinker^a^	366 (42.7)	3,137 (59.2)	<0.001
Metabolic status^b^			
Metabolic syndrome, *N* (%)	156 (18.2)	908 (17.1)	0.458
Increased blood pressure, *N* (%)	540 (62.9)	3,309 (62.5)	0.803
Impaired glucose tolerance, *N* (%)	162 (18.9)	934 (17.6)	0.378
Elevated triglyceride, *N* (%)	168 (19.6)	998 (18.8)	0.612
Low HDL-C, *N* (%)	157 (18.3)	670 (12.7)	<0.001
Abdominal obesity, *N* (%)	235 (27.4)	1,608 (30.4)	0.077

BMI, body mass index; HDL-C, high-density lipoprotein cholesterol.

^a^Data obtained using a questionnaire.

^b^Elevated blood pressure: systolic BP ≥130 mm Hg and/or diastolic BP ≥85 mm Hg. Impaired glucose tolerance: high fasting glucose (≥110 mg/dL). Elevated triglyceride levels: ≥150 mg/dL or low HDL-C: <40 mg/dL for men and <50 mg/dL for women. Abdominal obesity: waist circumstance of >85 cm for men and >90 cm for women.

Variables are expressed as mean (standard deviation), number (%), and median (25^th^ percentile, 75^th^ percentile). The differences between sleeping pill users and non-users were compared using unpaired *t*-tests and the Chi-squared test.

**Figure 1.**
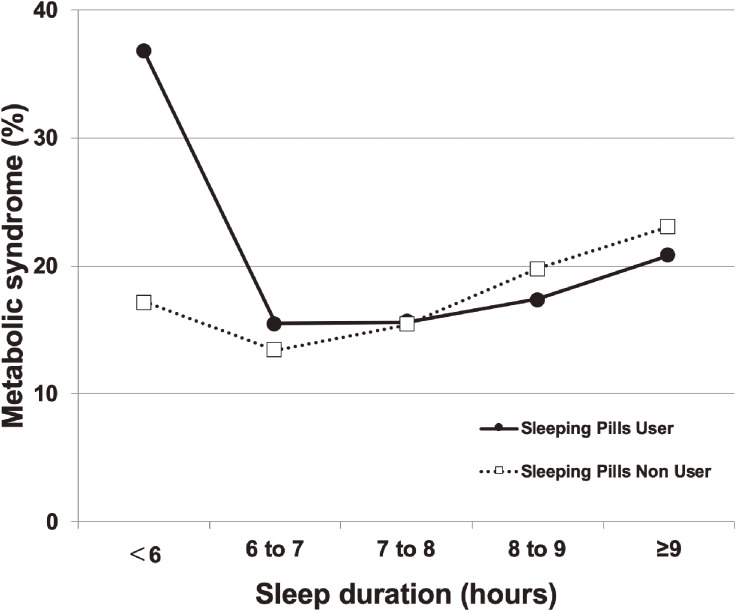
Association between sleep duration and metabolic syndrome in sleeping pill users and non-users.

**Figure 2.**
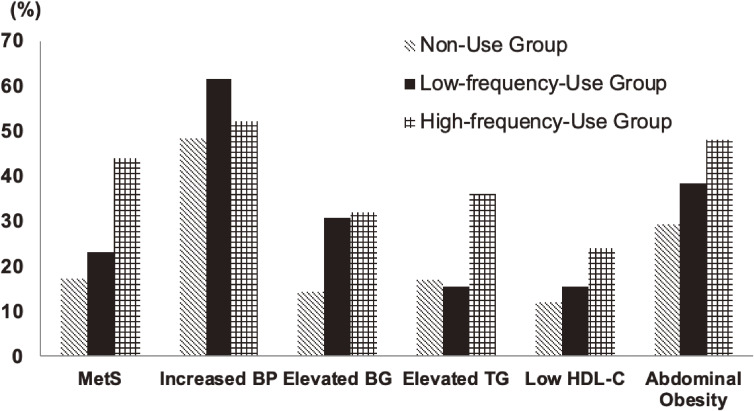
Association between the frequency of sleeping pill use and metabolic syndrome and metabolic components in individuals with short sleep duration. BP: blood pressure; BG: blood glucose; HDL-C: high-density lipoprotein cholesterol; MetS: metabolic syndrome; TG: triglyceride.

The associations between sleeping pill use, sleep duration, and MetS are depicted as an inverted J-shaped curve in the sleeping pill user group and as a J-shaped curve in the non-user group in Figure 1. The effects of sleeping pill use on the association between sleep duration and MetS after adjusting for various confounders are described using odds ratios (ORs) in Table 2. Increase in the prevalence of MetS in individuals with short sleep durations and a high frequency of sleeping pill use is shown in Figure 2. The associations between the prevalence of increased BP, elevated BG, low HDL-C levels, and abdominal obesity and the frequency of sleeping pill use were similar to the association with MetS.

**Table 2.** The association between sleeping pills, sleep duration, and metabolic syndrome

	Sleeping pills		Adjusted OR (95% CI)

	Non-user, *n* (%)	User, *n* (%)	
Metabolic syndrome			
Sleep duration, hours per night			
<6	71/412 (17.2)	14/38 (36.8)	3.08 (1.29–7.34)
6 to 7	152/1,132 (13.4)	17/110 (15.5)	1.18 (0.65–2.16)
7 to 8	278/1,803 (15.4)	38/244 (15.6)	1.15 (0.77–1.71)
8 to 9	253/1,281 (19.8)	45/258 (17.4)	0.98 (0.68–1.41)
≥9	154/667 (23.1)	42/208 (20.2)	1.11 (0.73–1.70)

CI, confidence interval; OR, odd ratio.

The relationship between the sleeping pills user and MetS was assessed with multivariate logistic regression analysis. The multivariable model was used, adjusted for age, gender, current smoker ex-smoker, current drinker, past medical history of myocardial infarction, stroke, and cancer, marriage status (yes or no), education status (<18 years or ≥18 years), and depressive symptoms (yes or no).

## DISCUSSION

We investigated the association between sleeping pill use and MetS in this population-based, cross-sectional study. The main findings were as follows: (1) Sleeping pill users with a short sleep duration had a 3-fold higher chance of having MetS than non-users with a short sleep duration; (2) The frequency of sleeping pill use was positively associated with the prevalence of MetS and its metabolic components among sleeping pill users with a short sleep duration.

Sleeping pills can exacerbate or inhibit atherosclerosis, directly or indirectly. The direct effects of sleeping pills include increased appetite, low sympathetic nerve activity, low insulin sensitivity, and low non-insulin-mediated glucose disposal via the stimulation of GABA receptors and central and peripheral benzodiazepine receptors.^28^^–^^32^ Sleeping pills relieve symptoms of insomnia and anxiety and extend the sleep duration of subjects. These effects might indirectly lead to a decrease in the risks of MetS and CVD, but these relationships might be very complex in a clinical setting.

In our study, the use of sleeping pill was associated with the high prevalence of MetS in the general population, after adjusting for multiple variables. Furthermore, the positive association between the frequency of the use of sleeping pills and the prevalence of MetS in those with a short sleep duration was observed. Short sleep durations were found to increase the prevalence of obesity, hypertension, dyslipidemia, MetS, and diabetes mellitus in most cross-sectional and longitudinal studies, independent of insomnia and the use of sleeping pills.^5^^,^^9^^,^^10^^,^^33^^–^^35^ The intervention of sleep restriction (<4 to 5 h/night) significantly reduced insulin sensitivity and impaired glucose tolerance thorough multiple mechanisms.^36^ In another healthy cohort, it was observed that even minor reductions in sleep duration lead to dramatic changes in body weight and metabolic parameters, including leptin levels.^37^ Our study, consistent with previous studies, showed that a short sleep duration increases the prevalence of MetS. The detailed pathogenesis of sleeping pill-induced MetS in individuals with short sleep durations remains to be elucidated. We examined the characteristics of sleeping pill users stratified into the sleep duration categories to explore some potential explanations for the result that only those using sleeping pills with a short sleep duration had an increased prevalence of MetS. As shown in eTable 1, depressive symptoms were highly prevalent in those with a short sleep duration. Given that there is the relation between depressive disorder and MetS and the patients with depressive disorder might have a poor response to sleeping pills, the high prevalence of depressive symptoms might be associated with the higher chance of MetS in those with a short sleep duration. However, the result remains the same, after adjusting the variable, and other uncontrolled confounding variables might affect the result (data not shown). Our study showed the unique association between abdominal obesity and sleeping pill. In Table 1, sleeping pill user seems not to be associated with abdominal obesity. However, in Figure 2, the proportion of abdominal obesity was higher in high-frequency-use group. There might be uncontrolled confounding variables affecting the association between the frequency of sleeping pill use and the prevalence of abdominal obesity. The primary sleep disorders, such as obstructive sleep apnea, were independently associated with an increased prevalence of MetS representing abdominal obesity and might also have a poor response to sleeping pills.^38^ Obstructive sleep apnea might affect the association, through the worsening of hypoxemia and the stimulation of sympathetic nervous system. However, we could not assess their effect appropriately, because of the data deficiency regarding these variables. Further studies are needed to determine the association. As sleeping pills might have a prominent negative effect with regard to MetS, it is important to pay attention to metabolic status and atherosclerosis risk, particularly in sleeping pill users with a short sleep duration.

There are several potential limitations to this study. Though this cohort study had a large sample size, the proportion of sleeping pill users with a short sleep duration was small. Therefore, our results need to be validated through another larger external cohort. This study did not assess data such as objective sleep duration, the dose of sleeping pills, and the classes of sleeping pills (eg, benzodiazepine, non-benzodiazepine, and a new generation of sleeping pills, including melatonin and orexin receptor antagonists). Recent studies have reported that the new generation of sleeping pills may have direct beneficial effects on metabolic function^39^^,^^40^; however, owing to our study period, it is likely that our results mainly involve the use of benzodiazepines or non-benzodiazepines. Moreover, there may be other potentially important underlying confounders, such as primary sleep disorders and diseases causing lowered activity, that were not considered in this study. Primary sleep disorders that can affect insomnia, such as sleep apnea and restless legs syndrome, might affect the association between the frequency of sleeping pill use and the prevalence of MetS and abdominal obesity and the association between sleeping pill use, sleep duration, and MetS.

Furthermore, this study also lacked longitudinal observation. The frequency of sleeping pill use may change during long-term follow-up, and our analysis was not adjusted for these variables. A case-control trial is warranted to investigate the prognostic effect of sleeping pill use after short-term and long-term follow-up.

### Conclusion

Sleeping pill users with a short sleep duration had a 3-fold higher chance of having MetS than non-users with a short sleep duration. The frequency of sleeping pill use was positively associated with the prevalence of MetS and its components among sleeping pill users with a short sleep duration. Hence, it is important to pay attention to the metabolic status and atherosclerosis risk among sleeping pill users with a short sleep duration.

## APPENDIX A. SUPPLEMENTARY DATA

eTable 1. The characteristics of sleeping pill users stratified into the sleep duration categories
