# Supplementary material for: The Association Between Sleeping Pill Use and Metabolic Syndrome in an Apparently Healthy Population in Japan: JMS-II Cohort Study
Source: J Epidemiol. 2022 Mar 5;32(3):145–50. doi: 10.2188/jea.JE20200361 (PMC8824657; doi:10.2188/jea.JE20200361)
Supplement: Supplementary file 1 [file je-32-145-s001.pdf]

eTable 1. The characteristics of sleeping pill users stratified into the sleep duration categories

	Sleep durations, hours				
	<6 (N=38)	6 to 7 (N=110)	7 to 8 (N=244)	8 to 9 (N=258)	≥9 (N=208)
Male, N (%)	10, 26.3	28, 25.5	68, 27.9	74, 28.7	87, 41.8
Age, years	61.8±12.3	65.1±9.7	67.1±9.6	70.9±9.1	74.9±9.0
Marriage status, N (%) [a]	26, 68.4	84, 78.5	187, 76.6	191, 74.6	137, 65.9
Education, N (%) [a]	23, 60.5	66, 60.0	138, 57.0	115, 44.6	68, 33.5
Depressive symptoms, N (%) [a]	15, 39.5	25, 22.7	66, 27.0	41, 15.9	54, 26.0
Past Medical History [a]					
Stroke, N (%)	3, 8.3	4, 3.6	8, 3.3	8, 3.1	13, 6.4
Myocardial infarction, N (%)	2, 5.6	2, 1.9	8, 3.3	8, 3.1	9, 4.5
Cancer, N (%)	1, 2.8	8, 7.3	17, 7.1	25, 9.8	19, 9.5
Smoking [a]					
Current	2, 5.3	13, 11.8	14, 5.7	23, 8.9	14, 6.7
EX	11, 28.9	21, 19.1	51, 20.9	52, 20.2	59, 28.4
Never	25, 65.8	76, 69.1	179, 73.4	183, 70.9	135, 64.9
Current Drinker [a]	17, 44.7	54, 49.1	108, 44.3	111, 43.0	76, 36.5

[a] Data were obtained using a questionnaire

Variables are expressed as mean ± standard deviation (SD) and number (%).
